# MicroRNA-196a/-196b regulate the progression of hepatocellular carcinoma through modulating the JAK/STAT pathway via targeting SOCS2

**DOI:** 10.1038/s41419-019-1530-4

**Published:** 2019-04-15

**Authors:** Weihua Ren, Shuangting Wu, Yabin Wu, Tan Liu, Xingpeng Zhao, Yawei Li

**Affiliations:** 1 Medical Diagnostic Centre, Luoyang Central Hospital Affiliated to Zhengzhou University, Luoyang, China; 2 Department of Anesthesiology, Luoyang Central Hospital Affiliated to Zhengzhou University, Luoyang, China; 3 Department of Orthopedics, Luoyang Central Hospital Affiliated to Zhengzhou University, Luoyang, China

## Abstract

microRNAs (miRNAs) play essential roles in progression of hepatocellular carcinoma (HCC). However, the roles of miR-196a and miR-196b as well as mechanism in HCC progression remain poorly understood. The expressions of miR-196a, miR-196b and suppressor of cytokine signaling 2 (SOCS2) were measured in HCC tissues and cells by quantitative real-time polymerase chain reaction or immunohistochemistry. HCC progression was investigated by cell proliferation, glycolysis, cycle, clones, apoptosis, and necrosis. The interaction between SOCS2 and miR-196a or miR-196b was explored by luciferase activity and RNA immunoprecipitation analyses. The expressions of proteins in Janus kinase (JAK)/signal transducer and activator of transcription (STAT) pathway were measured by western blot. A xenograft model was established to investigate the roles of miR-196a or miR-196b in vivo. We found that miR-196a and miR-196b were highly expressed in HCC tissues and cells. High expression of miR-196a or miR-196b was correlated with tumor size, tumor-node-metastasis stage, lymph node metastasis, albumin–bilirubin grade and poor 5-year survival. Knockdown of miR-196a or miR-196b suppressed cell proliferation, glycolysis, cell cycle process, colony formation but induced apoptosis or necrosis in HCC cells. SOCS2 was targeted by miR-196a and miR-196b and its interference ablated abrogation of miR-196a or miR-196b-mediated inhibitory effect on HCC progression. SOCS2 was negatively associated with activation of the JAK/STAT pathway. Besides, knockdown of miR-196a or miR-196b limited xenograft tumor growth by blocking the JAK/STAT pathway. We concluded that downregulation of miR-196a or miR-196b inhibited HCC progression through regulating the JAK/STAT pathway via targeting SOCS2, providing novel targets for prognosis and therapeutics of HCC.

## Introduction

Hepatocellular carcinoma (HCC) is one of the most common malignant tumors and the third leading cause of cancer death with rising incidence worldwide^1^. Despite great advances in diagnosis and treatment of HCC, the recurrence rate remains more than 80% and the survival rate of patients is still unsatisfactory^2^. Hence, it is urgent to explore novel strategies for diagnosis and therapeutics of HCC.

microRNAs (miRNAs) are a class of small noncoding RNAs with ~23 nucleotides in length, which play essential roles in development and progression of HCC^3^. miR-196 has been suggested to have an impact on the outcome of development and therapeutics of cancers^4^. Moreover, accruing works have indicated miR-196 as an oncogene to promote progression of various cancers, including glioblastoma, breast cancer, oral cancer, and digestive tract cancers^5–8^. The miR-196 family consists of miR-196a and miR-196b sharing regulatory efficacy^9^. Previous study has reported that miR-196a is notably upregulated in HCC tissues compared with that in matched nontumor samples^10^. Furthermore, miR-196b has been suggested to be highly expressed in HCC tissues and associated with HCC risk factors^11^. However, little is known about whether and how miR-196a and miR-196b could regulate HCC progression.

The Janus kinase (JAK)/signal transducer and activator of transcription (STAT) pathway has been regarded as one of the main molecular pathways in HCC progression^12^. STAT proteins, including STAT1-6, exhibit promoting or suppressive effect on antiviral response, inflammation, and tumorigenesis in liver, one of which, STAT5, could promote liver damage^13^. Suppressor of cytokine signaling (SOCS) family, such as SOCS1, SOCS2, and SOCS3, has been indicated as key negative regulator of the JAK/STAT signaling pathway in HCC^14–16^. The available evidence indicated the importance of SOCS2 in various diseases and cancers by regulating biological processes through addressing the JAK/STAT pathway or other signaling^17^. Hence, we hypothesized that miR-196a and miR-196b might regulate progression of HCC by targeting SOCS2. In the present study, we measured the expression of miR-196a and miR-196b and explored their roles as well as underlying mechanism in HCC progression.

## Materials and methods

### Patients and tissue samples

A total of 72 patients with HCC who had not received any chemotherapy, radiotherapy, or related treatment before surgery were recruited from Luoyang Central Hospital Affiliated to Zhengzhou University. Written informed consent was obtained from all participants and the work was accepted by the Research Ethics Committee of Luoyang Central Hospital Affiliated to Zhengzhou University. Paired tumor and peri-tumor samples collected from patients were collected for hematoxylin and eosin (H&E) staining or immediately stored at −80 °C until used. The clinicopathological characteristics of patients are shown in Table 1. The expression data of miR-196a and miR-196b were provided from database of YM500v3 (http://driverdb.tms.cmu.edu.tw/ym500v3/). The 5-year survival rate was investigated in every group. The correlation between clinicopathological characteristics of patients and miR-196a or miR-196b expression is presented in Tables 2 and 3. The liver damage was investigated according to the albumin–bilirubin (ALBI) score^18^.

**Table 1:** The clinic-pathological factors of 72 HCC patients

Clinicopathological feature	Number	% of patients
Age		
<55 years	40	55.5
≥55 years	32	44.5
Gender		
Female	28	38.9
Male	44	61.1
Tumor size		
>5 cm	49	68.1
≤5 cm	23	31.9
AFP (μg/L)		
Normal	34	47.2
Abnormal	38	52.3
HBsAg		
Present	52	72.2
Absent	20	27.8
Lymph node metastasis		
Yes	20	27.8
No	52	72.2
TNM stage		
I–II	28	38.9
III–IV	44	61.1
ALBI grade		
1	28	38.9
2	42	58.3
3	2	2.8

AFP: alpha-fetoprotein; HBsAg: hepatitis B surface antigen;

TNM: tumor-node-metastasis; ALBI: albumin–bilirubin.

**Table 2:** Correlation between miR-196a expression and clinicopathological characteristics of 72 HCC Patients

Clinicopathological feature	miR-196a expression		*P* value
	High *n* (%)	Down *n* (%)	
Age			>0.05
<55 years	23 (57.5)	17 (42.5)	
≥55 years	17 (53.1)	15 (46.9)	
Gender			>0.05
Female	17 (60.7)	11 (39.3)	
Male	23 (52.3)	21 (47.7)	
Tumor size			<0.05
>5 cm	30 (61.2)	19 (38.8)	
≤5 cm	10 (43.5)	13 (56.5)	
AFP (μg/L)			>0.05
Normal	19 (55.9)	15 (44.1)	
Abnormal	21 (55.3)	17 (44.7)	
HBsAg			>0.05
Present	27 (51.9)	25 (48.1)	
Absent	13 (65)	7 (35)	
Lymph node metastasis			<0.05
Yes	12 (60)	8 (40)	
No	28 (53.8)	24 (46.2)	
TNM stage			<0.05
I–II	12 (42.9)	16 (57.1)	
III–IV	28 (63.6)	16 (36.4)	
ALBI grade			<0.05
1	13 (46.4)	15 (53.6)	
2	25 (59.5)	17 (40.5)	
3	2 (100)	0 (0)	

AFP: alpha-fetoprotein; HBsAg: hepatitis B surface antigen;

TNM: tumor-node-metastasis; ALBI: albumin–bilirubin.

**Table 3:** Correlation between miR-196b expression and clinicopathological characteristics of 72 HCC Patients

Clinicopathological feature	miR-196b expression		*P* value
	High *n* (%)	Down *n* (%)	
Age			>0.05
<55 years	21 (52.5)	19 (47.5)	
≥55 years	18 (56.2)	14 (43.8)	
Gender			>0.05
Female	16 (57.1)	12(42.9)	
Male	23 (52.3)	21 (47.7)	
Tumor size			<0.05
>5 cm	33 (67.3)	16 (32.7)	
≤5 cm	6 (26.1)	17 (73.9)	
AFP (μg/L)			>0.05
Normal	17 (50)	17 (50)	
Abnormal	22 (57.9)	16 (42.1)	
HBsAg			>0.05
Present	29 (55.8)	23 (44.2)	
Absent	10 (50)	10 (50)	
Lymph node metastasis			<0.05
Yes	13 (65)	7 (35)	
No	26 (50)	26 (50)	
TNM stage			<0.05
I–II	10 (35.7)	18 (44.3)	
III–IV	29 (65.9)	15 (34.1)	
ALBI grade			<0.05
1	12 (42.9)	16 (57.1)	
2	25 (59.5)	17 (40.5)	
3	2 (100)	0 (0)	

AFP: alpha-fetoprotein; HBsAg: hepatitis B surface antigen;

TNM: tumor-node-metastasis; ALBI: albumin–bilirubin.

### Cell culture and transfection

The normal hepatic cell line (LO2) and four HCC cell lines (SMMC-7721, MHCC97-H, Hep3B, and HepG2) were obtained from American Tissue Culture Collection (Manassas, VA, USA). All cells were maintained in Dulbecco’s Modified Eagle Medium (Gibco, Carlsbad, CA, USA) containing 10% fetal bovine serum (Gibco), 100U/ml penicillin and 100 μg/ml streptomycin (Invitrogen, Carlsbad, CA, USA) at 37 °C in a humidified atmosphere with 5% CO_2_ during the study.

miR-196a mimic (miR-196a), miR-196b mimic (miR-196b), miRNA negative control (miR-NC), miR-196a inhibitor (anti-miR-196a), miR-196b inhibitor (anti-miR-196b), miRNA inhibitor negative control (anti-miR-NC), small interfering RNA (siRNA) against SOCS2 (si-SOCS2), siRNA negative control (si-NC), pcDNA, or SOCS2 overexpression vector (SOCS2) were synthesized by Genepharma (Shanghai, China). Cell transfection was performed by using Lipofectamine 3000 (Invitrogen) according to the manufacturer’s instructions. Transfection efficacy was evaluated after 24 h of transfection.

### Quantitative real-time polymerase chain reaction (qRT-PCR)

Total RNA was isolated from tissues or cells by using TRIzol reagent (Invitrogen) following the manufacturer’s instructions. The TaqMan microRNA Reverse Transcription Kit (Applied Biosystems, Foster City, CA, USA) or M-MLV Reverse Transcription Kit (Invitrogen) was used for synthesis of complementary DNA (cDNA). The diluted cDNA was amplified using SYBR green (Applied Biosystems) with the following amplification protocol: 95 °C for 10 min, 40 cycles of 95 °C for 15 s, and 60 °C for 1 min. The expressions of miR-196a, miR-196b, and SOCS2 were normalized by U6 small RNA or β-actin with 2^−ΔΔCt^ method^19^. All primers were obtained from Sangon (Shanghai, China): miR-196a (Forward, 5′- ACCTGCGTAGGTAGTTTCATGT-3′ Reverse, 5′-CGTCA GAAGGAATGATGCACAG-3′), miR-196b (Forward, 5′-TAGGTACCACTTTATC CCGTTCACCA-3′ Reverse, 5′-ATCTCGAGGCAGGGAGAGAGGAATAA-3′), U6 (Forward, 5′-CTCGCTTCGGCAGCACATATACT-3′ Reverse, 5′-CGCTTCACG AATTTGCGTGT-3′), SOCS2 (Forward, 5′-GGAACGGCACTGTTCACCTTTA-3′ Reverse, 5′-AGCCTACAGAGATGCTGCAGAGA-3′), β-actin (Forward, 5′-CAGC CTTCCTTCTTGGGTAT-3′ Reverse, 5′-TGGCATAGAGGTCTTTACGG-3′).

### Cell proliferation

Cell proliferation was investigated by the 3-(4,5-dimethyl-2-thiazolyl)-2,5-diphenyl-2-H-tetrazolium bromide (MTT) assay. SMMC-7721 or HepG2 cells were seeded into 96-well plates at a density of 5000 cells per well. Each group was prepared in triplicate. Cells were interacted with MTT solution (Thermo Fisher, Wilmington, DE, USA) for 4 h at 0, 24, 48, or 72 h. Following the removal of the supernatant, 100 μl dimethylsulfoxide (Thermo Fisher) was added to dissolve formazan. The absorbance at 570 nm was detected using a microplate reader (Bio-Rad, Hercules, CA, USA).

### Measurement of glucose consumption and lactate production

SMMC-7721 and HepG2 cells were seeded in 96-well plates overnight. After 72 h, cells were washed three times in phosphate buffer saline (Gibco), and then collected for analyses of glucose consumption and lactate production via using the Glucose Uptake Colorimetric Assay Kit or Lactate Assay Kit (Sigma, St. Louis, MO, USA) according to the manufacturer’s instructions, respectively. The concentrations of glucose consumption and lactate production were measured by using a microplate reader and normalized by the total protein concentration determined via the bicinchoninic acid (BCA) assay kit (Thermo Fisher).

### Flow cytometry

Flow cytometry was conducted to measure cell apoptosis or necrosis. After 72 h, transfected cells were washed with PBS. Cells were resuspended in binding buffer and double stained with the Annexin V-fluorescein isothiocyanate (FITC)/propidium iodide (PI) apoptosis detection kit (Sigma) according to the manufacturer’s instructions. The apoptotic or necrotic cells were analyzed through a flow cytometer (Becton Dickinson, Franklin Lakes, NJ, USA) with CellQuest software.

### Western blot

Cells or tissues were lysed in RIPA lysis buffer (Thermo Fisher) containing 1% protease inhibitor (Sigma). After centrifuged at 16,000 × *g* for 20 min at 4 °C, total proteins were quantified by the BCA protein assay kit and then denatured at 98 °C for 10 min. Proteins were separated by SDS-PAGE gel electrophoresis and transferred to polyvinylidene difluoride membranes (Millipore, Billerica, MA, USA), followed by blocked with 5% nonfat milk in Tris-buffer saline containing 0.1% Tween 20 (TBST) for 1 h at room temperature. Subsequently, membranes were incubated with primary antibodies (1:1000 dilution, Cell Signaling Technology, Danvers, MA, USA) against B-cell lymphoma-2 (Bcl-2, #4223), Bcl-2 associated X proteins (Bax, #5023), SOCS2 (#2779), STAT5 (#25656), phosphorylation of STAT5 (p-STAT5, #4322), JAK2 (#3230), p-JAK2 (#3771), or β-actin (#4970) overnight at 4 °C. β-actin was regarded as loading control in this study. After washed with TBST for three times, membranes were incubated with horseradish peroxidase-conjugated secondary antibodies (#7074, 1:10000 dilution, Cell Signaling Technology) for 2 h at room temperature. The protein signals were visualized via using enhanced chemiluminescence chromogenic substrate (Thermo Fisher) and analyzed with Image Lab software (Bio-Rad).

### Luciferase activity assay

The putative binding sites of 3′ untranslated regions (3′-UTR) sequences of SOCS2 and miR-196a or miR-196b were predicted by using TargetScan Release 7.2 (http://www.targetscan.org/vert_72/). pGL3 vectors (Promega, Madison, WI, USA) were used to synthesize wide-type (SOCS2-WT) or mutant-type (SOCS2-MUT) luciferase reporter vectors containing wild-type or mutant binding sites, respectively. Luciferase reporter vectors and miR-196a, miR-196b or miR-NC were cotransfected in HepG2 cells using Lipofectamine 3000 according to the manufacturer’s protocols. After the transfection for 48 h, luciferase activity was analyzed by using the luciferase assay kit (Promega) and normalized to Renilla luciferase activity.

### RNA immunoprecipitation (RIP)

The RIP assay was conducted in HepG2 cells by using the RNA-binding protein immunoprecipitation kit (Millipore) according to the manufacturer’s protocols. HepG2 cells transfected with miR-196a, miR-196b, or miR-NC were lysed in RIP buffer with anti-Ago2- or IgG-bound magnetic beads. The mRNA level of SOCS2 enriched on beads was measured by qRT-PCR after treatment of TRIzol reagent.

### Immunohistochemistry

Tumor and peri-tumor tissues were fixed with 4% polyoxymethylene (Sigma), embedded in paraffin and sectioned to 4 μm thick. Following blocking endogenous peroxidase using 3% H_2_O_2_ (Sigma), sections were incubated with primary antibodies against SOCS2 (ab74533, 1:100 dilution, Abcam, Cambridge, UK) for 2 h and incubated with anti-rabbit IgG for 30 min, followed by stained with diaminobenzidine and hematoxylin (Sigma). The positive cells were detected under a light microscopy (Olympus).

### Murine xenograft model

BALB/c nude mice (male, four-week-old) were obtained from Vital River Laboratory Animal Technology (Beijing, China) and housed in specific pathogen-free conditions with a 12 h light/dark cycle and free access to water and food. Every experiment was made to minimize animals (*n* = 7 per group) under the approval of the Animal Research Committee of Luoyang Central Hospital Affiliated to Zhengzhou University. HepG2 cells were transfected with the lentiviral vectors with anti-miR-196a (Lenti-anti-miR-196a), anti-miR-196b (Lenti-anti-miR-196b), anti-miR-NC (Lenti-anti-miR-NC), or negative control (Lenti-NC) constructed by GeneCopoeia (Rockville, MD, USA). Stably transfected cells (5 × 10^6^) were subcutaneously injected into the nude mice. Tumor volumes were measured every week for 5 weeks with the formula: volume (mm^3^) = width^2^ × length/2. After 5 weeks following the inoculation, the mice were killed and tumor samples were collected and weighted. The collected tumor tissues were used for further molecular studies. Cell apoptosis and proliferation in tumor tissues were measured by terminal deoxynucleotidyl transferase dUTP nick-end labeling (TUNEL) staining with in situ cell death detection kit (Roche, Mannheim, Germany) or Ki67 immunohistochemistry with anti-Ki67 antibody (ab16667, 1:1000 dilution, Abcam) following the manufacturer’s instructions as previous study^20^.

### Statistical analysis

The data were presented as the mean ± standard deviation (S.D.) from three independent experiments. The survival curve was generated via Kaplan–Meier method. The statistical differences between groups were investigated by Student’s *t* test or one-way analysis of variance (ANOVA) using SPSS 18.0 software (SPSS, Inc., Chicago, IL, USA). *P* < 0.05 was regarded as statistically significant.

## Results

### miR-196a and miR-196b expressions are enhanced and SOCS2 level is decreased in HCC tissues and cells

**Fig. 1:**
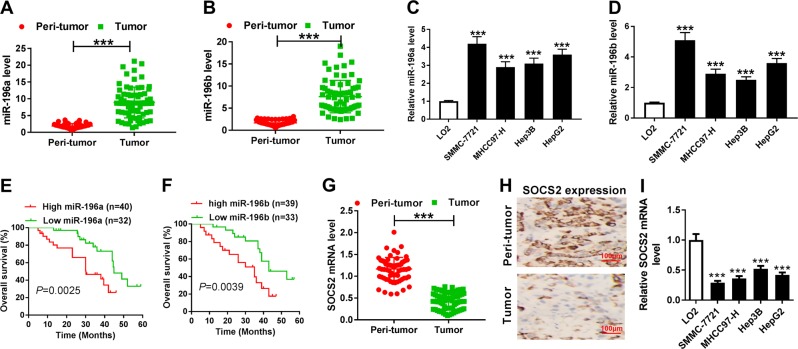
The expressions of miR-196a, miR-196b and SOCS2 in HCC tissues and cells. **a**, **b** The expressions of miR-196a and miR-196b were measured in tumor and peri-tumor tissues of HCC patients by qRT-PCR. **c**, **d** The expressions of miR-196a and miR-196b were detected in HCC cells by qRT-PCR. **e**, **f** The overall survival rate of patients was analyzed in low miR-196a, high miR-196a, low miR-196b, or high miR-196b group by Kaplan–Meier method. **g**–**i** The expression of SOCS2 was measured in tissues or cells by qRT-PCR or immunohistochemistry. ***P < 0.001

The morphology investigation of HCC and peri-tumor tissues using H&E staining displayed that stained HCC tissues showed typical HCC morphology with many formed balloon-like lesions and the peri-tumor tissues exhibited normal liver cell morphology (Fig. S1). Bioinformatics analysis suggested that miR-196a and miR-196b were highly expressed in HCC tissues compared with that in normal tissues by YM500v3 (Fig. S2A and S2B). To explore the roles of miR-196a and miR-196b in HCC progression, their expression levels were measured in HCC tissues. Compared with peri-tumor group, HCC tissues showed higher expression levels of miR-196a and miR-196b (Fig. 1a, b). Similarly, their levels were also increased in HCC cells compared with those in control group (Fig. 1c, d). Furthermore, patients were classified as high expression and low expression group according to the mean value of miR-196a or miR-196b expression level, named as high miR-196a expression (*n* = 40) and low miR-196a expression (*n* = 32) or high miR-196b expression (*n* = 39) and low miR-196b expression (*n* = 33) group, respectively. Results showed that high expression of miR-196a or miR-196b was associated with tumor size, tumor-node-metastasis stage, lymph node metastasis, and ALBI grade (Tables 2, 3 and Fig. S2C–F). In addition, high expression of miR-196a or miR-196b displayed lower overall survival rate (*P* = 0.0025 or 0.0039, respectively) (Fig. 1e, f). Besides, the expression of SOCS2 mRNA and protein were significantly decreased in HCC compared with that in corresponding control (Fig. 1g–i).

### Knockdown of miR-196a or miR-196b inhibits progression of HCC cells

**Fig. 2:**
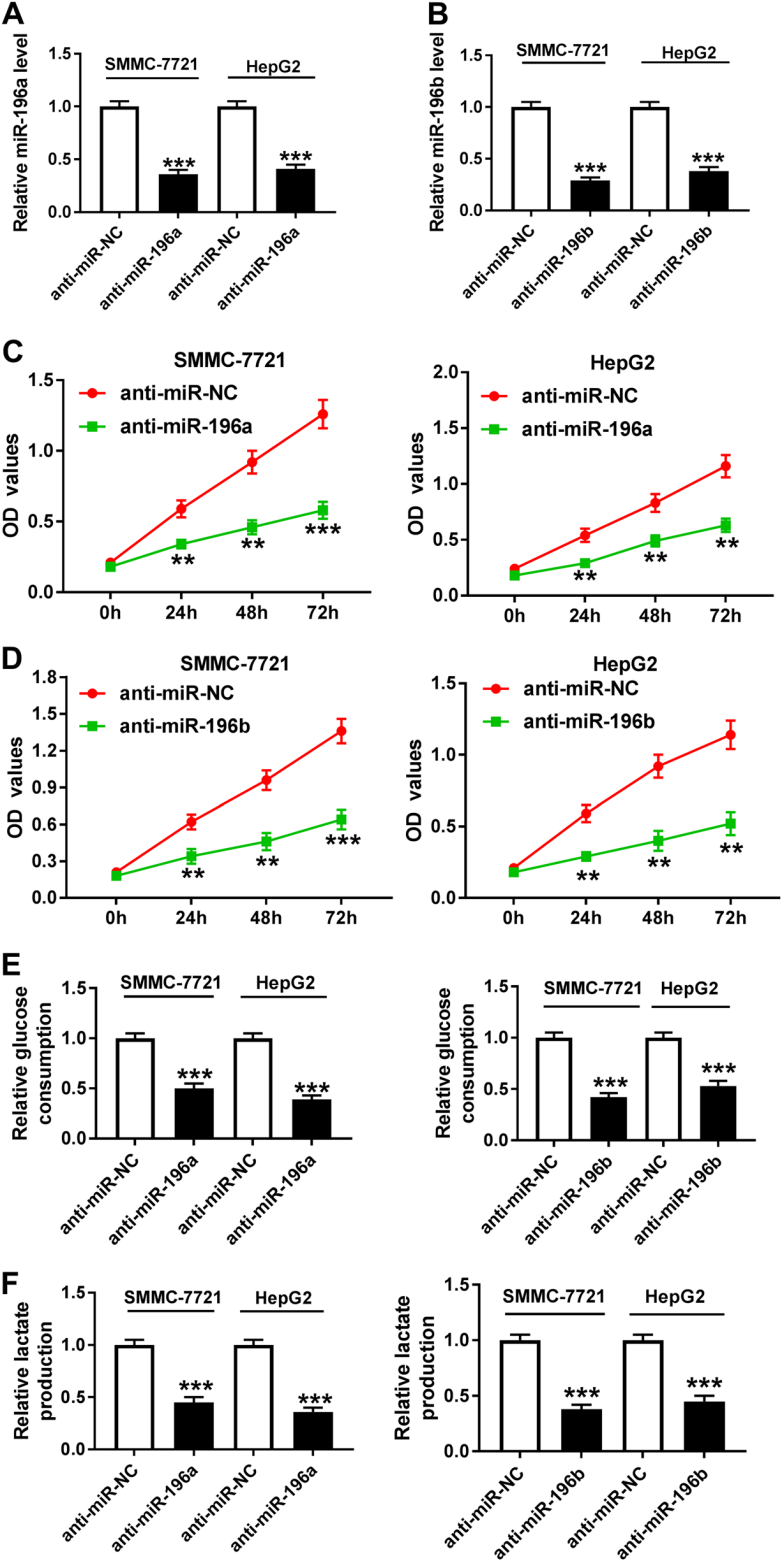
The effect of miR-196a or miR-196b knockdown on cell proliferation and glycolysis in HCC cells. SMMC-7721 and HepG2 cells were transfected with anti-miR-196a, anti-miR-196b, or their corresponding anti-miR-NC. Their expression levels (**a**, **b**), cell proliferation (**c**, **d**), glucose consumption and lactate production (**e**, **f**) were measured in the two cells by qRT-PCR, the MTT assay, or the special commercial kit. **P < 0.01, ***P < 0.001

**Fig. 3:**
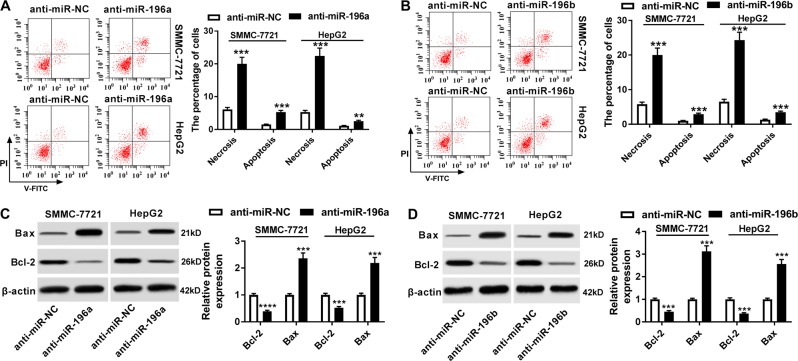
The effect of miR-196a or miR-196b depletion on cell apoptosis or necrosis in HCC cells. SMMC-7721 and HepG2 cells were treated as described in Fig. 2. **a**, **b** Cell apoptosis or necrosis was analyzed in transfected cells by flow cytometry. **c**, **d** The expressions of apoptosis-related proteins were measured in SMMC-7721 and HepG2 cells by western blot. ***P < 0.001, ****P < 0.0001

To investigate the potential roles of miR-196a and miR-196b in HCC, SMMC-7721 and HepG2 cells with higher abundances of miR-196a and miR-196b were transfected with anti-miR-196a, anti-miR-196b, or anti-miR-NC. The analysis of transfection efficacy showed that the abundances of miR-196a and miR-196b were effectively reduced in the two cells after transfection of anti-miR-196a or anti-miR-196b (Fig. 2a, b). Cell function analysis showed that knockdown of miR-196a or miR-196b suppressed HCC progression by inhibiting cell proliferation, glucose consumption and lactate production (Fig. 2c–f), arresting cell at G0/G1 stage (Fig. S3A and S3B) and promoting apoptosis or necrosis through regulating Bcl-2 and Bax (Fig. 3a–d). In addition, by infecting lentiviral vectors with anti-miR-196a (LV-anti-miR-196a), anti-miR-196b (LV-anti-miR-196b), or anti-miR-NC (LV-anti-miR-NC), the number of clones was obviously decreased in SMMC-7721 and HepG2 cells (Fig. S3C and S3D).

### miR-196a and miR-196b regulate HCC progression by targeting SOCS2

**Fig. 4:**
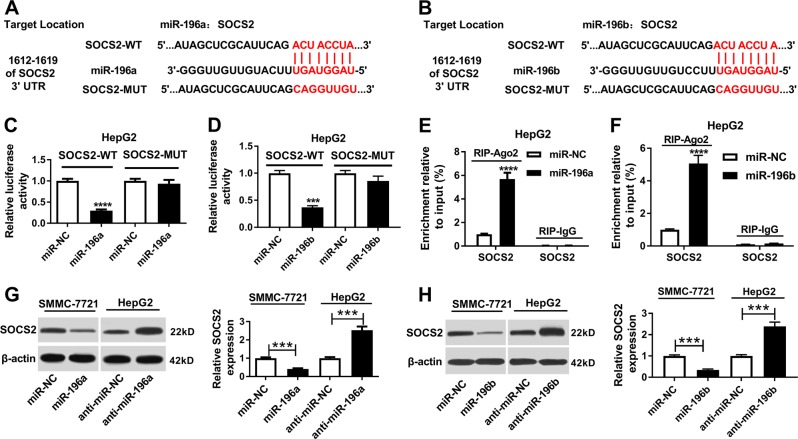
The association of SOCS2 and miR-196a or miR-196b. **a**, **b** The potential binding sites of SOCS2 and miR-196a or miR-196b were predicted by TargetScan Release 7.2. **c**, **d** Luciferase activity was analyzed in HepG2 cells cotransfected with SOCS2-WT or SOCS2-MUT and miR-196a, miR-196b or miR-NC. **e**, **f** The enrichment of SOCS2 was detected in HepG2 cells transfected with miR-196a, miR-196b or miR-NC after Ago2-RIP. **g**, **h** The expression of SOCS2 protein was detected in the two cells transfected with miR-196a, miR-196b, anti-miR-196a, anti-miR-196b, or their corresponding negative control by western blot. ***P < 0.001, ****P < 0.0001

To explore the potential mechanism underlies miR-196a or miR-196b participating in progression of HCC, the promising targets were wanted in HCC cells. Bioinformatics analysis provided the potential targets by TargetScan Release 7.2 (Table 4). Therein, SOCS2 with the potential binding sites is an important target involved in pathogenesis of HCC (Fig. 4a, b). To validate this prediction, luciferase activity and RIP analyses were conducted into HepG2 cells. Results showed that overexpression of miR-196a or miR-196b significantly reduced luciferase activity in HepG2 cells transfected with SOCS2-WT, whereas the efficacy was lost in term of SOCS2-MUT (Fig. 4c, d). Moreover, the enrichment of SOCS2 abundance was notably elevated in miR-196a or miR-196b-transfected group compared with that in miR-NC group after Ago2 RIP, while IgG failed to show efficacy of enrichment (Fig. 4e, f). Meanwhile, SOCS2 displayed higher ability of miR-196a or miR-196b enrichment than SOCS1 (Fig. S4A and S4B). Moreover, western blot data showed that accumulation of miR-196a or miR-196b limited SOCS2 protein level, while deficiency of miR-196a or miR-196b played an opposite effect (Fig. 4g, h).

**Table 4:** The part of predict targets of miR-196a/b from TargetScan Release 7.2

miR	Target gene	Representative transcript	Gene name
miR-196a	SOCS2	ENST00000548537.1	Suppressor of cytokine signaling 2
	HMGA2	ENST00000403681.2	High mobility group AT-hook 2
	ING5	ENST00000313552.6	Inhibitor of growth family, member 5
	HOXC8	ENST00000040584.4	Homeobox C8
	FAM127A	ENST00000257013.7	Family with sequence similarity 127, member A
	GALC	ENST00000261304.2	Galactosylceramidase
	ABCB9	ENST00000280560.8	ATP-binding cassette, sub-family B (MDR/TAP), member 9
	MAP4K3	ENST00000263881.3	Mitogen-activated protein kinase kinase kinase kinase 3
miR-196b	SOCS2	ENST00000548537.1	Suppressor of cytokine signaling 2
	ABCB9	ENST00000280560.8	ATP-binding cassette, sub-family B (MDR/TAP), member 9
	MAP4K3	ENST00000263881.3	Mitogen-activated protein kinase kinase kinase kinase 3
	TMEM143	ENST00000293261.3	Transmembrane protein 143
	IQCJ-SCHIP1	ENST00000337808.6	IQCJ-SCHIP1 readthrough
	TBPL1	ENST00000237264.4	TBP-like 1
	SSR1	ENST00000244763.4	Signal sequence receptor, alpha
	SNAP91	ENST00000521485.1	Synaptosomal-associated protein, 91 kDa

**Fig. 5:**
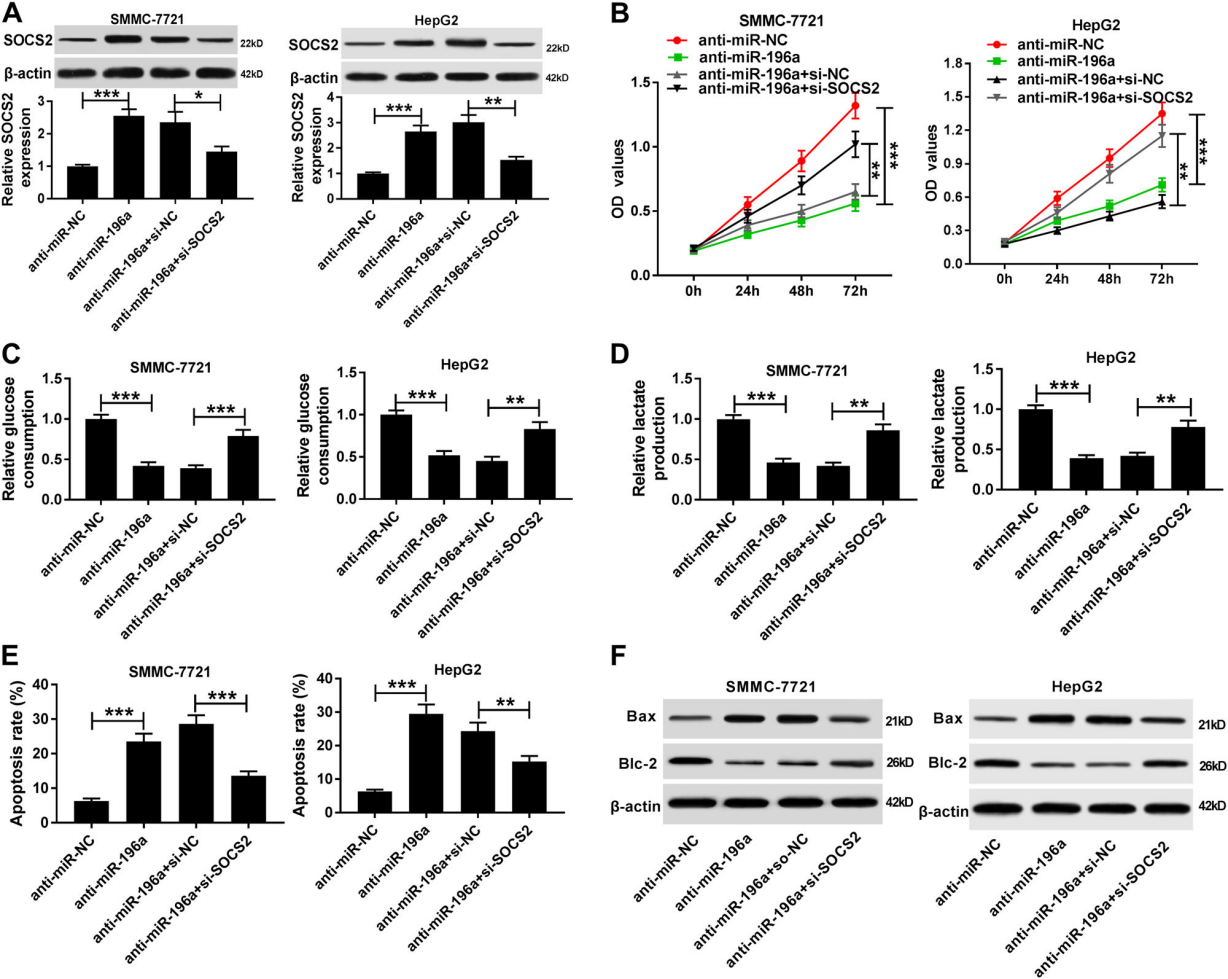
The regulatory role of SOCS2 interference in miR-196a-mediated HCC progression. SMMC-7721 and HepG2 cells were transfected with anti-miR-NC, anti-miR-196a, or along with si-NC or si-SOCS2. The SOCS2 protein level **a**, cell proliferation **b**, glucose consumption **c**, lactate production **d**, and apoptosis **e**, **f** were detected in the transfected cells by western blot, the MTT assay, the commercial kit, or flow cytometry. *P < 0.05, **P < 0.01, ***P < 0.001

**Fig. 6:**
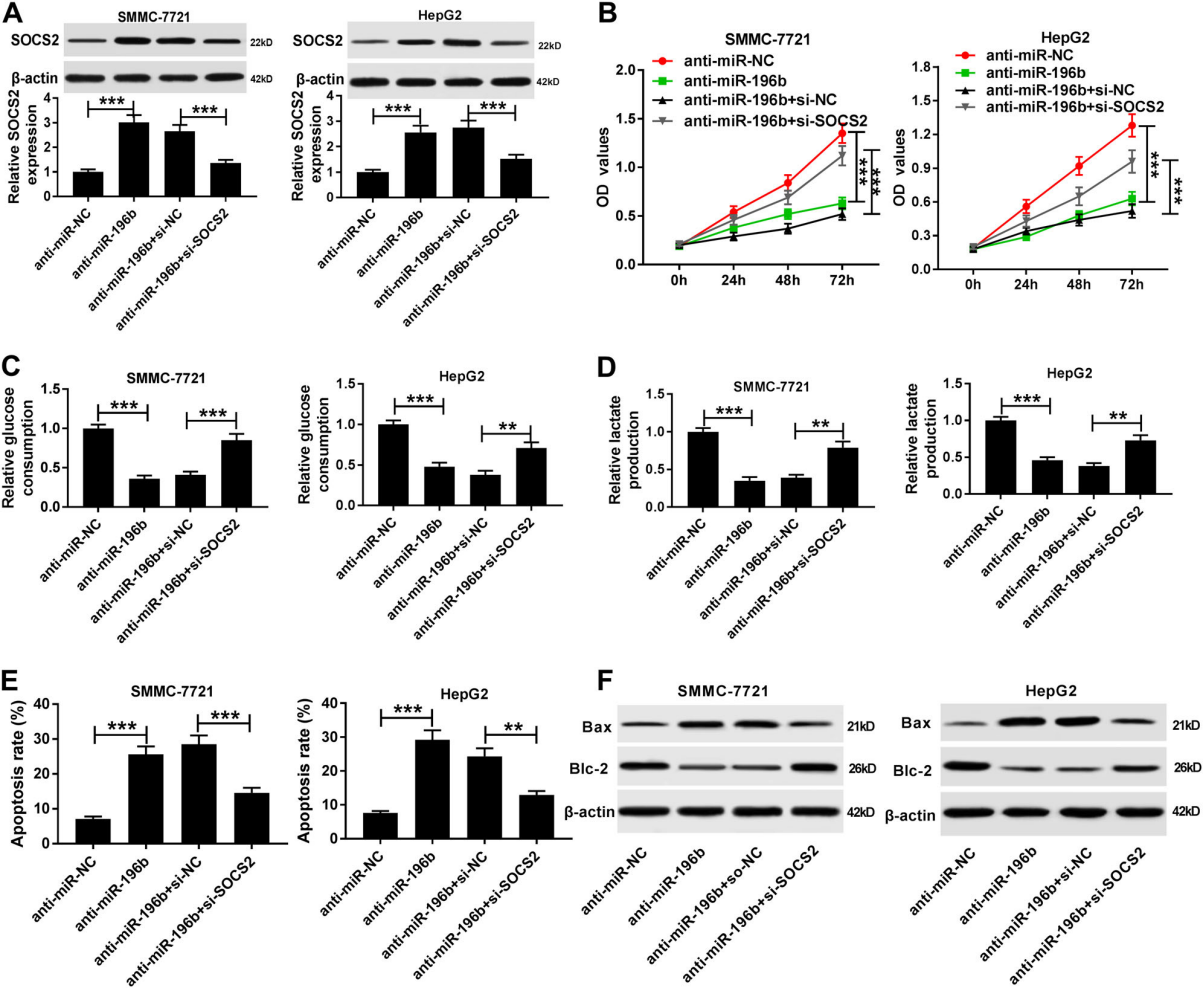
The restorative effect of SOCS2 silence in miR-196b-mediated HCC progression. SMMC-7721 and HepG2 cells were transfected with anti-miR-NC, anti-miR-196b, or along with si-NC or si-SOCS2. The SOCS2 protein abundance **a**, cell proliferation **b**, glucose consumption **c**, lactate production **d**, and apoptosis **e**, **f** were analyzed in the two transfected cells by western blot, the MTT assay, the commercial kit, or flow cytometry, respectively. **P < 0.01, ***P < 0.001

To investigate whether SOCS2 was required for miR-196a or miR-196b-mediated progression of HCC, SMMC-7721 and HepG2 cells were cotransfected with anti-miR-196a or anti-miR-196b and si-SOCS2, or si-NC. Transfection efficacy was validated in Figs. 5a and 6a. Moreover, recuse experiments displayed that knockdown of SOCS2 reversed downregulation of miR-196a or miR-196b-mediated inhibition of cell proliferation, glycolysis, cell cycle progress, colony formation, and apoptosis (Figs. 5b–f, 6b–f, Fig. S5A-D).

### Depletion of miR-196a or miR-196b inhibits HCC progression by regulating the JAK/STAT pathway

**Fig. 7:**
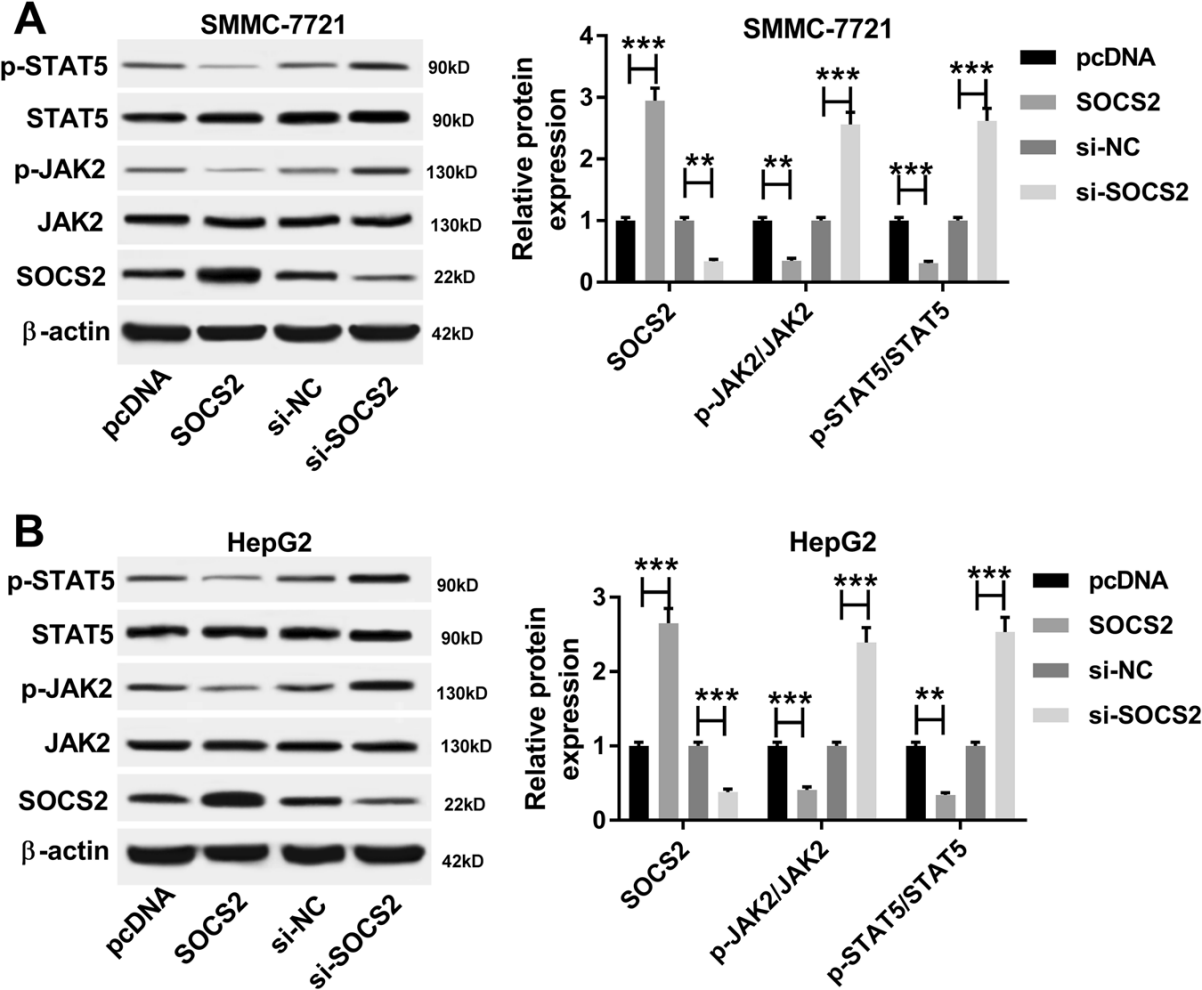
The effect of SOCS2 on the JAK/STAT pathway in HCC cells. **a**, **b** The expressions of p-STAT5, STAT5, p-JAJ2, JAK2, and SOCS2 protein were measured in SMMC-7721 and HepG2 cells transfected with SOCS2, pcDNA, si-SOCS2, or si-NC by western blot. **P < 0.01, ***P < 0.001

To explore the potential signaling pathway participating in HCC progression in this study, the expressions of p-STAT5, STAT5, p-JAK2, and JAK2 proteins were measured in SMMC-7721 and HepG2 cells transfected with SOCS2, pcDNA, si-SOCS2, or si-NC. As a result, the abundance of SOCS2 protein was effectively elevated by transfection of SOCS2 expression vector and reduced by introduction of si-SOCS2 compared with their corresponding control, respectively (Fig. 7a, b). Moreover, overexpression of SOCS2 inhibited phosphorylation of JAK2 and STAT5, whereas its knockdown promoted the expressions of p-JAK2 and p-STAT5 in SMMC-7721 and HepG2 cells (Fig. 7a, b).

**Fig. 8:**
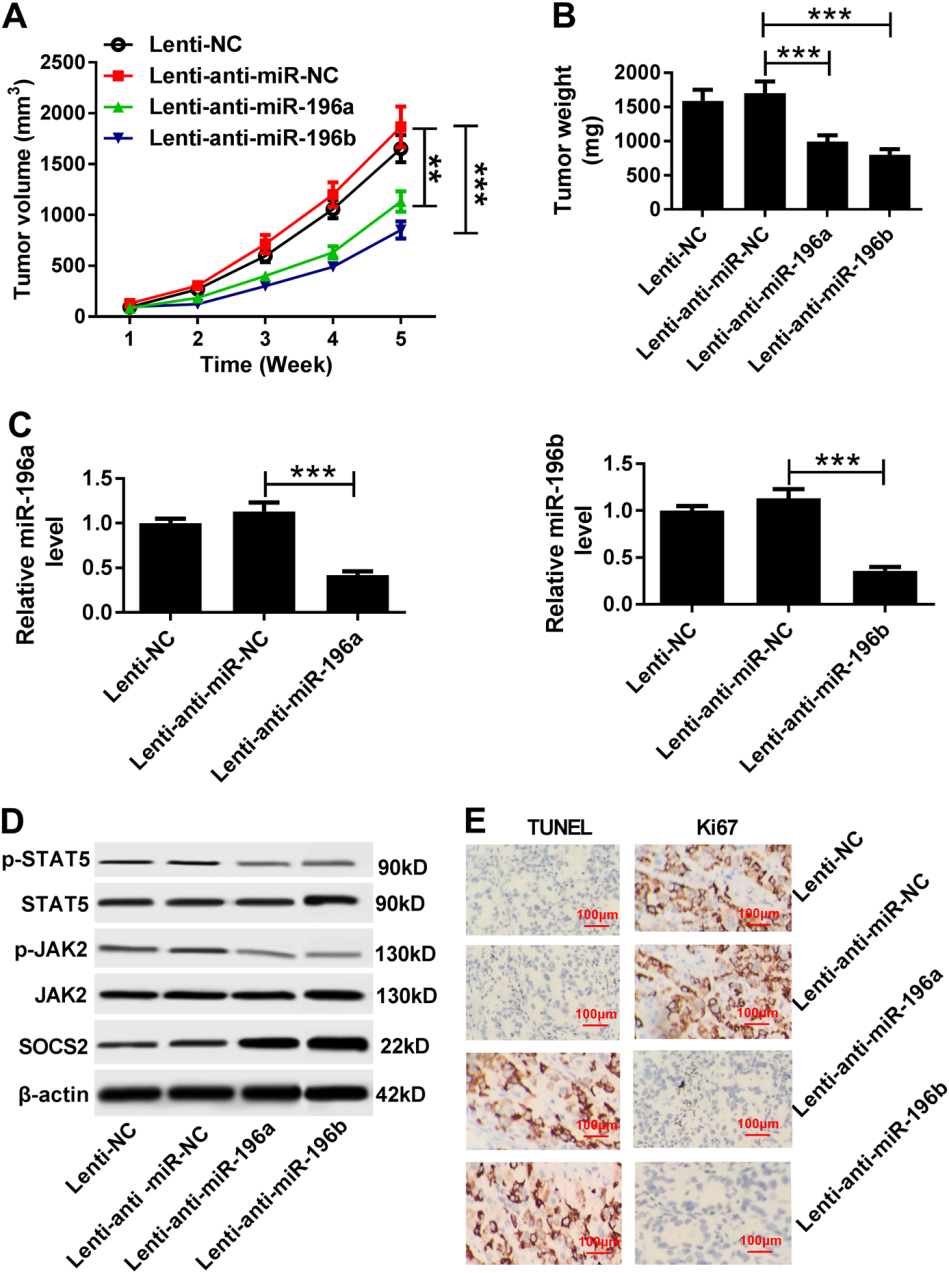
The effect of miR-196a or miR-196b abrogation on xenograft tumor growth. HepG2 cells were transfected with Lenti-NC, Lenti-anti-miR-NC, Lenti-anti-miR-196a, or Lenti-anti-miR-196b. Then stably transfected cells were subcutaneously injected into the nude mice. **a** Tumor volume was measured every week for 5 weeks. **b** Tumor weight was detected at ending point. **c** The expression of miR-196a or miR-196b was examined in tumor tissues by qRT-PCR. **d** The protein expressions in the JAK2/STAT5 pathway were analyzed in each group by western blot. **e** Cell apoptosis and proliferation were investigated in tumor tissues by TUNEL or Ki67 immunohistochemistry. **P < 0.01, ***P < 0.001

To further investigate the roles of miR-196a and miR-196b in HCC, the murine xenograft model was established by subcutaneously injecting with HepG2 cells infected with Lenti-NC, Lenti-anti-miR-NC, Lenti-anti-miR-196a, or Lenti-anti-miR-196b. Results displayed that knockdown of miR-196a or miR-196b decreased tumor volume and weight compared with treatment of Lenti-anti-miR-NC (Fig. 8a, b). Then the expression of miR-196a or miR-196b was measured in tumor tissues. Impaired expression of miR-196a or miR-196b was exhibited in Lenti-anti-miR-196a or Lenti-anti-miR-196b compared with that in Lenti-anti-miR-NC group, respectively (Fig. 8c). Subsequently, the abundances of p-STAT5, STAT5, p-JAK2, JAK2, and SOCS2 proteins were measured in tumor tissues. Results revealed that inhibition of miR-196a or miR-196b promoted SOCS2 protein level but blocked phosphorylation of JAK2 and STAT5 (Fig. 8d). In addition, tissues showed more apoptotic cells and less cell proliferation in Lenti-anti-miR-196a or Lenti-anti-miR-196b compared with that in Lenti-anti-miR-NC group, respectively (Fig. 8e). Besides, knockdown of miR-196a and miR-196b led to obvious decrease of Bcl-2 protein level and increase of Bax protein abundance in xenograft tumor tissues (Fig. S6A and S6B).

## Discussion

miRNAs have been reported to play pivotal roles in regulating development, diagnosis, and antitumor treatments of HCC^21^. In this study, we found that miR-196a and miR-196b expressions were enhanced in HCC tissues and cells, which is also in agreement with previous efforts^10,11^. This indicated that miR-196a and miR-196b might serve as oncogenes to promote HCC progression. However, the exact roles of miR-196a and miR-196b in HCC remain largely unknown. Here we explored the effect of miR-196a or miR-196b on HCC cell progression and first revealed the mechanism possibly associated with the SOCS2/JAK2/STAT5 signaling pathway.

The former finding has suggested that miR-196a knockdown inhibited cell proliferation, colony formation, migration, and invasion but induced cell apoptosis by targeting forkhead transcription factor O1 (FOXO1) in HCC^22^. Moreover, miR-196a was reported to contribute to cell proliferation through regulating cell cycle via targeting FOXO1 in HCC^23^. As for miR-196b, it was shown to predict poor prognosis and increase cell migration as well as invasion in HCC by regulating FOXP2^24^. Consistently with these reports, we also found that miR-196a and miR-196b knockdown could induce proliferation and apoptosis inhibition and indicate poor outcome of patients. Cell cycle process and colony formation are necessary for normal proliferation, while in this study the two miRNAs knockdown arrested cell cycle process and colony formation. Moreover, elevated glycolysis is an important progress in HCC development, which could support metabolic needs for rapid proliferation of cancer cells^25^. The intrinsic apoptosis pathway is addressed by pro-apoptotic protein Bax and antiapoptotic protein Bcl-2. The disturbance of Bcl-2/Bax balance contributes to apoptosis production. Our results revealed that knockdown of miR-196a and miR-196b inhibited glycolysis and Bcl-2 expression but promoted Bax level. These all findings suggested the poor outcomes of miR-196a and miR-196b in prognosis and progression of HCC.

Functional miRNAs are known to regulate targets expressions by binding their 3′-UTR sequences^26^. This research explored multiple targets of miR-196a and miR-196b and previous studies have validated the interaction between them and FOXO1, FOXP2, or lysosome-associated protein transmembrane-4β (LAPTM4B) in HCC^22–24,27^. SOCS proteins mediate inflammation and apoptosis via regulating immune homeostasis in human cancers. Although Zhao et al. have revealed SOCS2 as a target of miR-196b in human laryngeal squamous cell carcinoma^28^, here we focused on the interaction between miR-196a or miR-196b and SOCS2, which displayed higher miRNA-bound ability than SOCS1. Increasing evidences demonstrated that SOCS2 might function as a tumor suppressor in HCC by regulating tumor progression^29–32^. By luciferase activity and RIP assays we identified SOCS2 as a functional target of miR-196a and miR-196b in HCC cells. And recuse experiment by further SOCS2 silence uncovered that miR-196a and miR-196b regulated HCC progression by targeting SOCS2.

Active JAKs recruit STAT proteins to mediate gene transcription, participating in cell proliferation, apoptosis and glucose metabolism. SOCS2 along with other members of SOCS family are regarded as importantly negative regulator of the JAK/STAT pathway^33^. Although STAT3 signaling might be more important than STAT5 in HCC progression and miR-196b could activate STAT3 by targeting SOCS2 in macrophages^34,35^, previous work showed that importance of JAK2/STAT5 signaling in liver metabolism, liver diseases, and HCC^36^. Hence, we assumed JAK/STAT signaling was implicated in miR-196a- or miR-196b-mediated progression of HCC. This study uncovered that SOCS2 negatively regulated activation of the JAK/STAT pathway in HCC cells and miR-196a or miR-196b inhibition decreased HCC progression by regulating the SOCS2/JAK/STAT pathway revealed by a xenograft model.

In conclusion, high expressions of miR-196a and miR-196b were shown in HCC and predicted poor outcome of patients. Knockdown of miR-196a or miR-196b inhibited HCC progression, possibly by the SOCS2/JAK2/STAT5 pathway. This study indicates that miR-196a and miR-196b might serve as promising therapeutic therapy for HCC treatment.

## Supplementary information

Supplementary materials

Sup Figure 1

Sup Figure 2

Sup Figure 3

Sup Figure 4

Sup Figure 5

Sup Figure 6

supplementary figure legends

## Data Availability

The datasets from current study are available from the corresponding author on reasonable request.
