# Supplementary material for: MicroRNA-196a/-196b regulate the progression of hepatocellular carcinoma through modulating the JAK/STAT pathway via targeting SOCS2
Source: Cell Death Dis. 2019 Apr 15;10(5):333. doi: 10.1038/s41419-019-1530-4 (PMC6465376; doi:10.1038/s41419-019-1530-4)
Supplement: Supplementary file 1 — Supplementary materials [file 41419_2019_1530_MOESM1_ESM.docx]

**Materials and methods**

**Hematoxylin and eosin (H&E) staining**

HCC tissues and peri-tumor samples were fixed with 4% paraformaldehyde solution (Sigma, St. Louis, MO, USA) for 24 h and then dehydrated in graded ethanol, embedded in paraffin, and cut into 5 µm thick sections, followed by staining with hematoxylin and eosin and observed under a microscope (x 400, Olympus, Tokyo, Japan).

**Cell cycle and colony formation assays**

Flow cytometry was conducted to measure cell cycle. After transfection for 72h, cells were washed with PBS. For cell cycle assay, SMMC-7721 and HepG2 cells were fixed with 70% ethanol at 4^o^C overnight, incubated with RNase at 37^o^C, and then stained with propidium iodide (PI, Sigma), followed by detected using a flow cytometer (Becton Dickinson, Franklin Lakes, NJ, USA).

For colony formation assay, SMMC-7721 and HepG2 were infected with lentiviral vectors with anti-miR-196a (LV-anti-miR-196a), anti-miR-196b (LV-anti-miR-196b), anti-miR-NC (LV-anti-miR-NC), sh-NC or sh-COCS2 constructed by GeneCopoeia (Rockville, MD, USA). The infected cells were seeded into 6-well plates at a density of 500 cells per well and then cultured at 37°C for two weeks. Colonies were fixed with methanol (Sigma), stained with 0.01% crystal violet (Sigma), and then counted with an inverted microscope.

**RNA immunoprecipitation (RIP)**

RIP assay was conducted in HepG2 cells by using RNA-binding protein immunoprecipitation kit (Millipore, Billerica, MA, USA) according to the manufacturer’s protocols. HepG2 cells transfected with miR-196a, miR-196b or miR-NC were lysed in RIP buffer with anti-Ago2- or IgG-bound magnetic beads. The mRNA levels of SOCS1 and SOCS2 enriched on beads was measured by qRT-PCR after treatment of TRIzol reagent.
