# Supplementary figures and images for: MicroRNA-196a/-196b regulate the progression of hepatocellular carcinoma through modulating the JAK/STAT pathway via targeting SOCS2
Source: Cell Death Dis. 2019 Apr 15;10(5):333. doi: 10.1038/s41419-019-1530-4 (PMC6465376; doi:10.1038/s41419-019-1530-4)

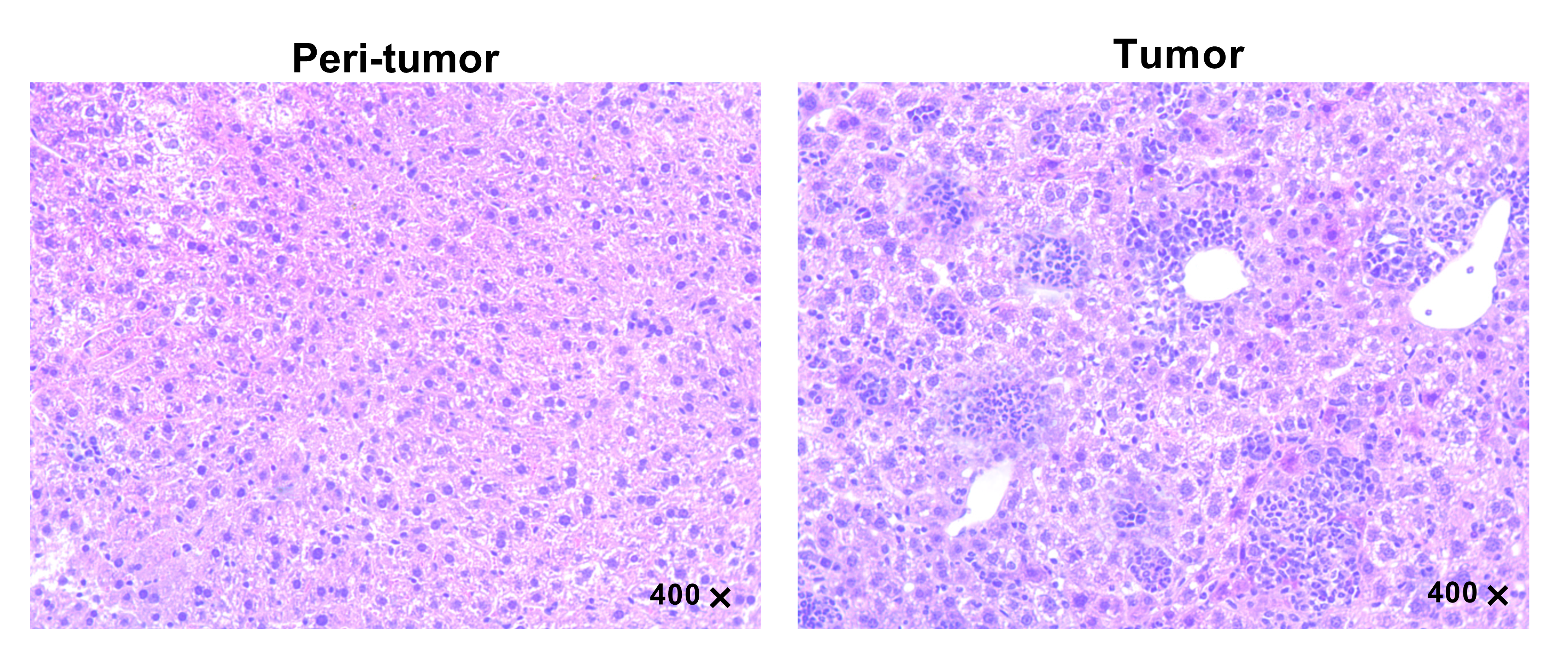

Supplement: Supplementary file 2 — Sup Figure 1 [file 41419_2019_1530_MOESM2_ESM.tif]

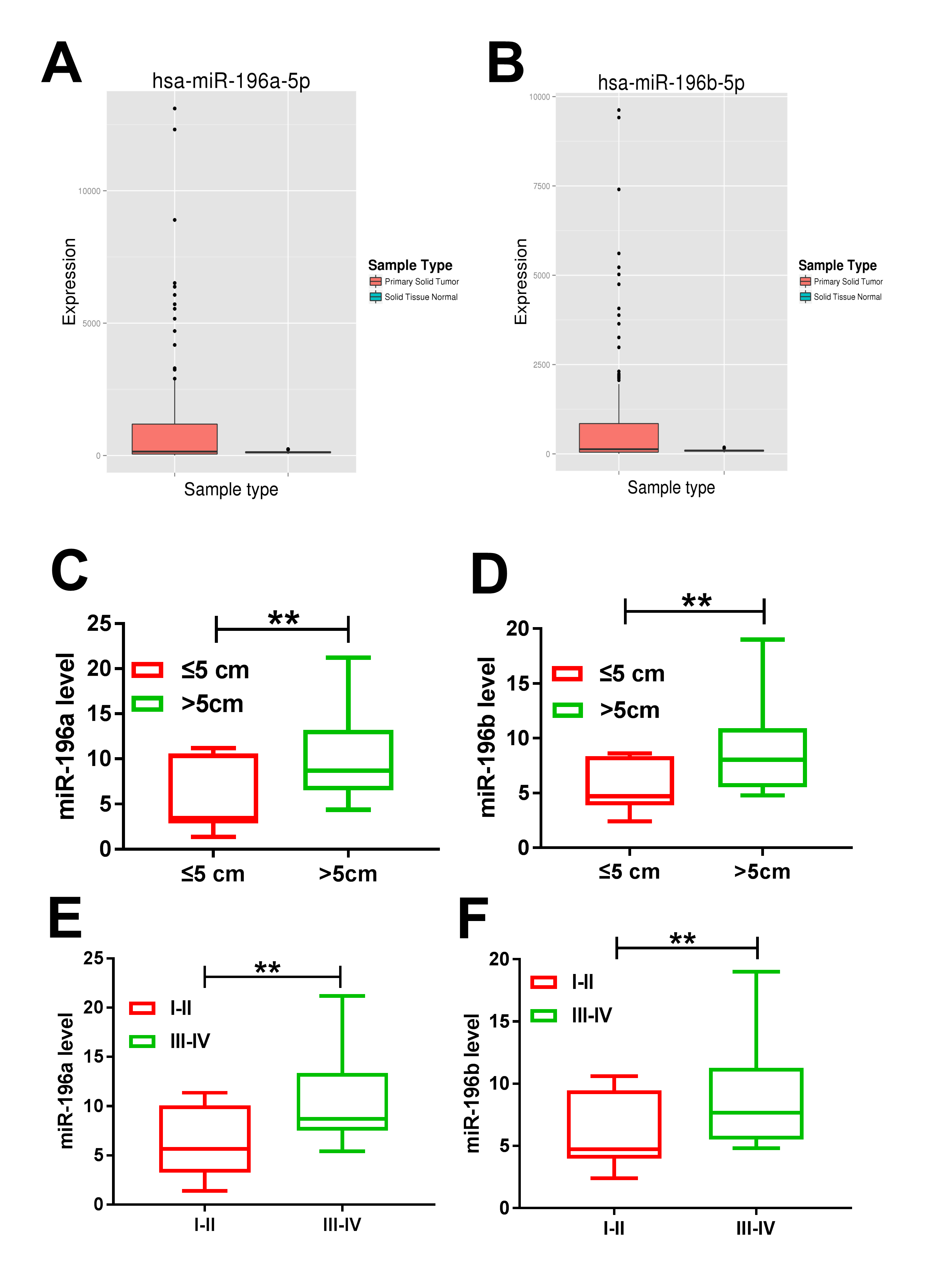

Supplement: Supplementary file 3 — Sup Figure 2 [file 41419_2019_1530_MOESM3_ESM.tif]

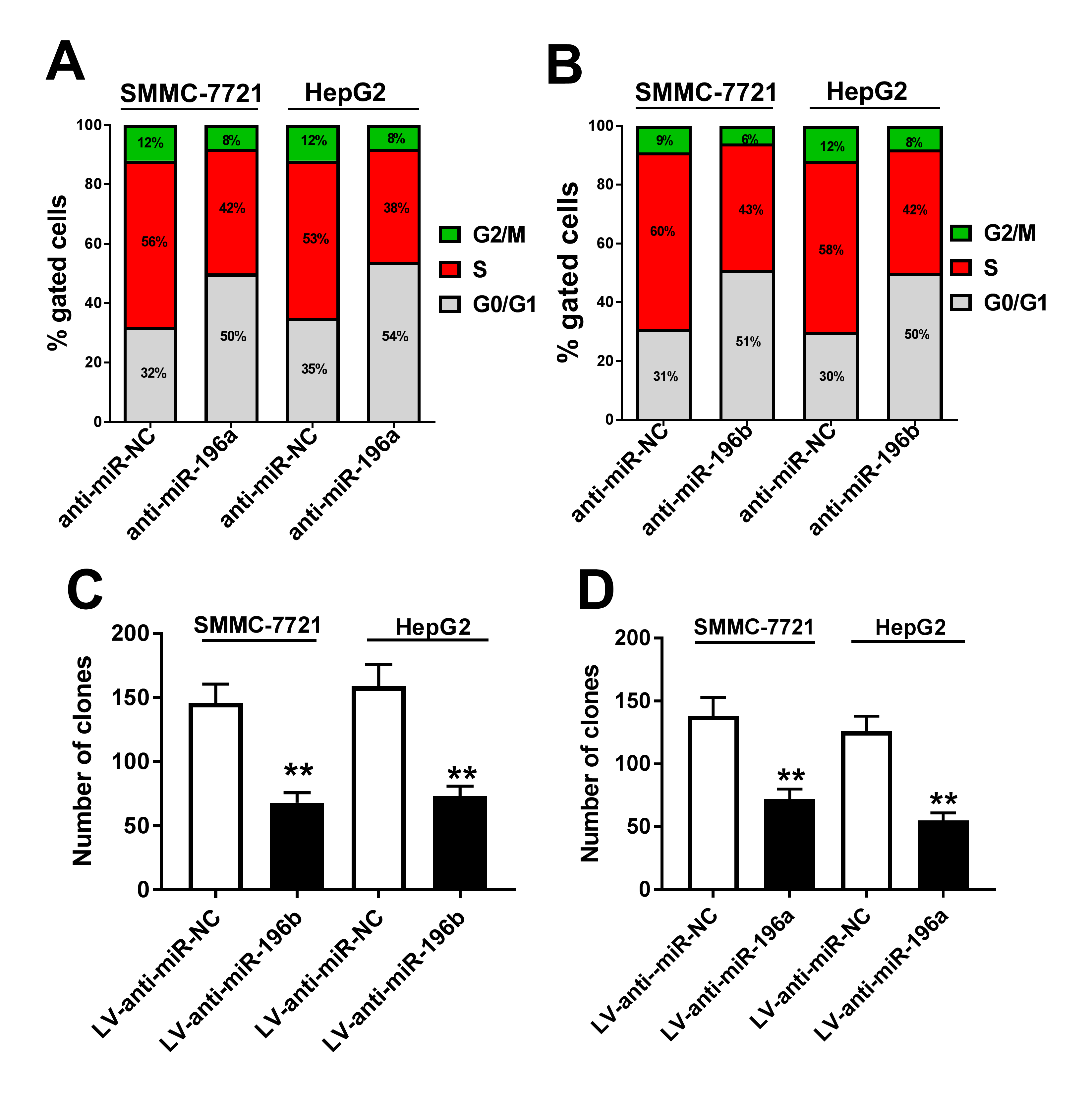

Supplement: Supplementary file 4 — Sup Figure 3 [file 41419_2019_1530_MOESM4_ESM.tif]

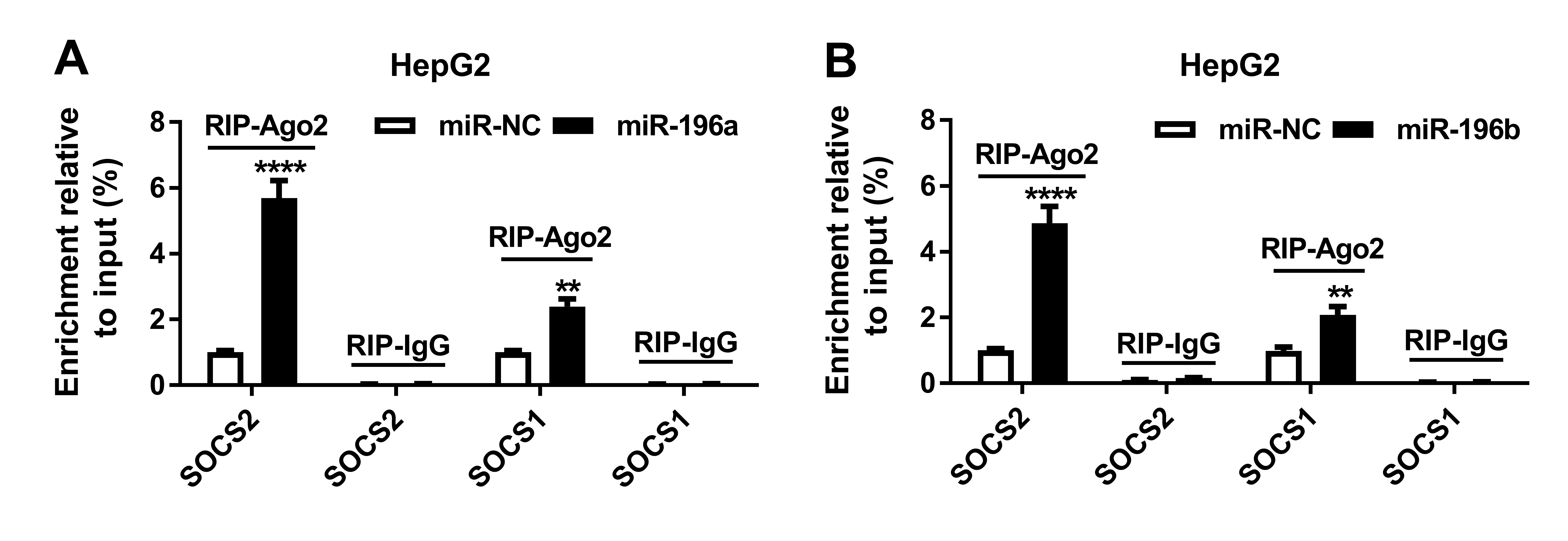

Supplement: Supplementary file 5 — Sup Figure 4 [file 41419_2019_1530_MOESM5_ESM.tif]

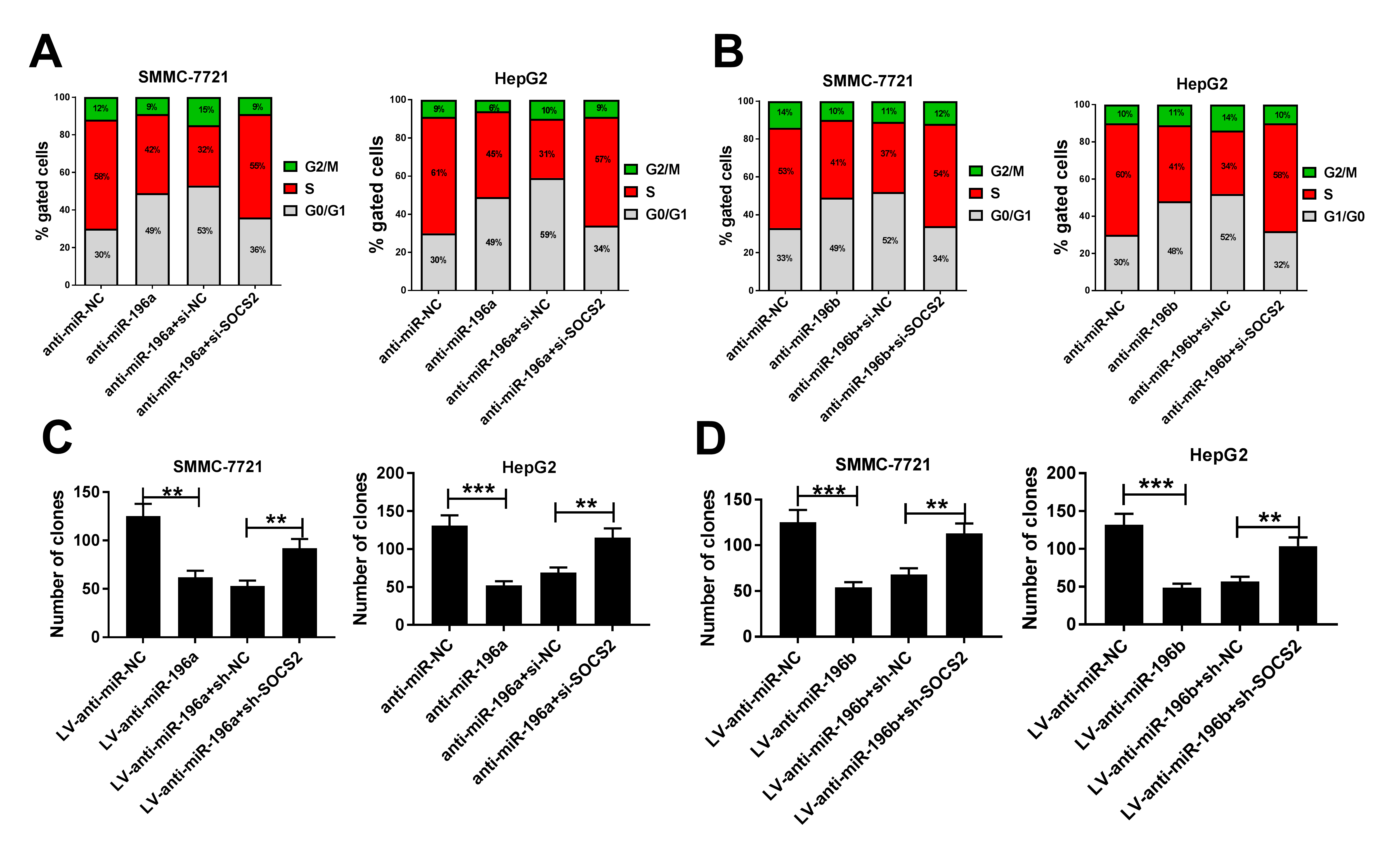

Supplement: Supplementary file 6 — Sup Figure 5 [file 41419_2019_1530_MOESM6_ESM.tif]

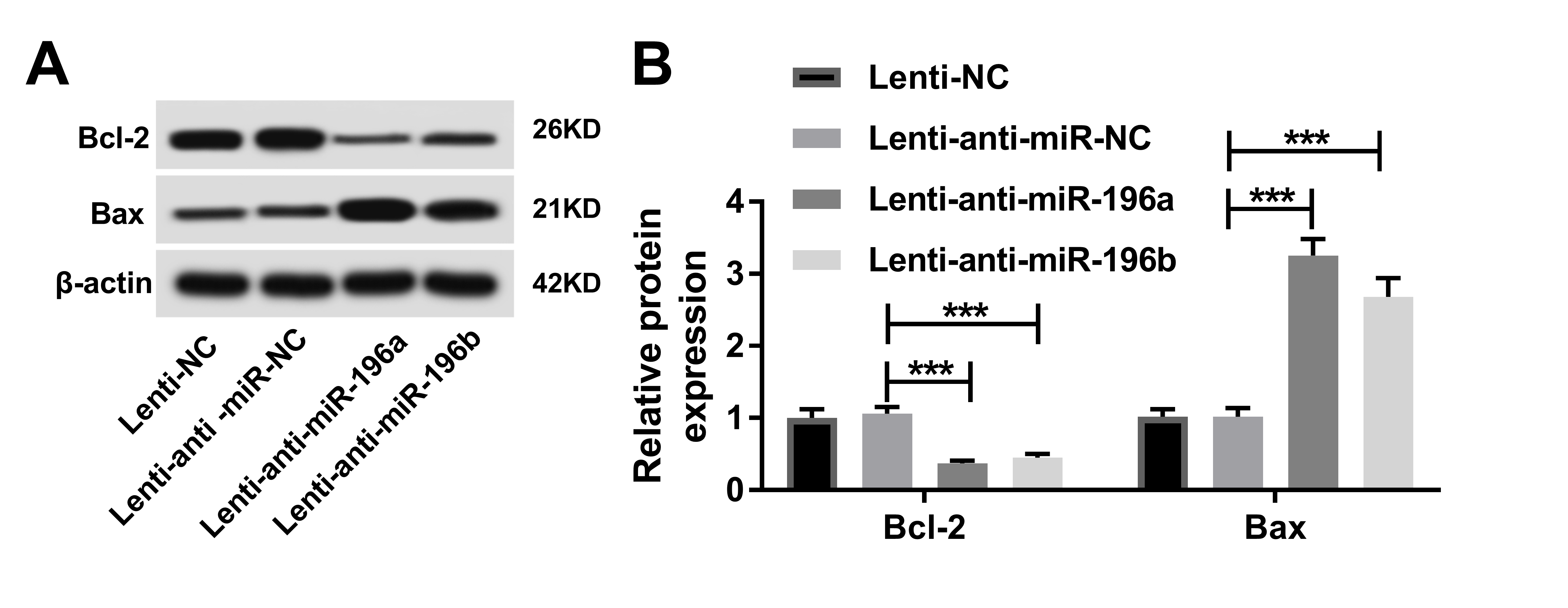

Supplement: Supplementary file 7 — Sup Figure 6 [file 41419_2019_1530_MOESM7_ESM.tif]
