# Supplementary material for: MicroRNA-196a/-196b regulate the progression of hepatocellular carcinoma through modulating the JAK/STAT pathway via targeting SOCS2
Source: Cell Death Dis. 2019 Apr 15;10(5):333. doi: 10.1038/s41419-019-1530-4 (PMC6465376; doi:10.1038/s41419-019-1530-4)
Supplement: Supplementary file 8 — supplementary figure legends [file 41419_2019_1530_MOESM8_ESM.docx]

**Supplementary figure legends**

**Supplementary Figure 1. The images of H&E staining in peri-tumor and tumor tissues.** The morphology of HCC and adjacent tissues was observed under a microscope (X 400).

**Supplementary Figure 2. The expressions of mir-196a and miR-196b in HCC.** (A and B) The expression data of miR-196a and miR-196b were provided by database of YM500v3. (C and D) The levels of miR-196a and miR-196b were detected in tumor size >5cm and ≤5cm group by qRT-PCR. (E and F) The abundances of miR-196a and miR-196b were examined in tumor-node-metastasis (TNM) stage I-II and III-IV group by qRT-PCR. ***P*<0.01.

**Supplementary Figure 3.** **The effect of** **miR-196a or miR-196b abrogation on cell cycle progress and colony formation in HCC cells.** (A and B) Cell cycle was measured in SMMC-7721 and HepG2 cells transfected with anti-miR-196a, anti-miR-196b or their corresponding anti-miR-NC by flow cytometry. (C and D) The numbers of clone were measured in the two cells infected with Lenti-anti-miR-NC, Lenti-anti-miR-196a or Lenti-anti-miR-196b. ***P*<0.01.

**Supplementary Figure 4. The association of SOCS1 or SOCS2 and miR-196a or miR-196b.** (A and B) The enrichment of SOCS1 and SOCS2 was measured in HepG2 cells transfected with miR-NC, miR-196a or miR-196b after Ago2-RIP. ***P*<0.01, *****P*<0.0001.

**Supplementary Figure 5. The effect of SOCS2 on miR-196a or miR-196b-mediated cell cycle progress and colony formation in HCC cells.** (A and B) Cell cycle was measured in SMMC-7721 and HepG2 cells transfected with anti-miR-NC, anti-miR-196b, miR-196b, or along with si-NC or si-SOCS2 by flow cytometry. (C and D) The numbers of clone were measured in the two cells infected with Lenti-anti-miR-NC, Lenti-anti-miR-196a, Lenti-anti-miR-196b or along with sh-NC or sh-SOCS2. ***P*<0.01, ****P*<0.001.

**Supplementary Figure 6. The effect of miR-196a or miR-196b knockdown on apoptosis in xenograft tumor tissues.** (A and B) The expression of apoptosis-related protein was measured in xenograft tumor tissues in each group. ****P*<0.001.
